# Supplementary figures and images for: Fusarium spp. Associated with Dendrobium officinale Dieback Disease in China
Source: J Fungi (Basel). 2022 Aug 29;8(9):919. doi: 10.3390/jof8090919 (PMC9504887; doi:10.3390/jof8090919)

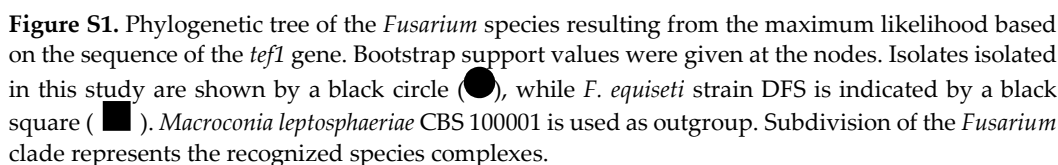

Supplement: Supplementary file 1 [file jof-08-00919-s001.zip › jof-1852407-supplementary.pdf]
